# Movement visualisation in virtual reality rehabilitation of the lower limb: a systematic review

**DOI:** 10.1186/s12938-016-0289-4

**Published:** 2016-12-19

**Authors:** Luara Ferreira dos Santos, Oliver Christ, Kedar Mate, Henning Schmidt, Jörg Krüger, Christian Dohle

**Affiliations:** 1 Rehabilitation Robotics Group (TU Berlin/ Fraunhofer IPK), Department of Industrial Automation Technology, Technische Universität Berlin, Pascalstr. 8-9, 10587 Berlin, Germany; 2 DFG Research Training Group Prometei, Technische Universität Berlin, Marchstr. 23, 10587 Berlin, Germany; 3 Institute Humans in Complex Systems, School of Applied Psychology, University of Applied Sciences and Arts Nortwestern Switzerland, Riggenbachstrasse 16, Olten, Switzerland; 4 School of Physical and Occupational Therapy, McGill University, 3654 Promenade Sir William Osler Montreal, Quebec, H3G 1Y5 Canada; 5 Rehabilitation Robotics Group (Fraunhofer IPK/ TU Berlin), Department of Automation Technology, Fraunhofer Institute for Production Systems and Design Technology (IPK), Pascalstr. 8-9, 10587 Berlin, Germany; 6 Department of Neurological Rehabilitation, MEDIAN Klinik Berlin-Kladow, Kladower Damm 223, 14089 Berlin, Germany; 7 Center for Stroke Research Berlin, Charité-University Medicine Berlin, Charitéplatz 1, 10117 Berlin, Germany

## Abstract

**Background:**

Virtual reality (VR) based applications play an increasing role in motor rehabilitation. They provide an interactive and individualized environment in addition to increased motivation during motor tasks as well as facilitating motor learning through multimodal sensory information. Several previous studies have shown positive effect of VR-based treatments for lower extremity motor rehabilitation in neurological conditions, but the characteristics of these VR applications have not been systematically investigated. The visual information on the user’s movement in the virtual environment, also called movement visualisation (MV), is a key element of VR-based rehabilitation interventions. The present review proposes categorization of Movement Visualisations of VR-based rehabilitation therapy for neurological conditions and also summarises current research in lower limb application.

**Methods:**

A systematic search of literature on VR-based intervention for gait and balance rehabilitation in neurological conditions was performed in the databases namely; MEDLINE (Ovid), AMED, EMBASE, CINAHL, and PsycInfo. Studies using non-virtual environments or applications to improve cognitive function, activities of daily living, or psychotherapy were excluded. The VR interventions of the included studies were analysed on their MV.

**Results:**

In total 43 publications were selected based on the inclusion criteria. Seven distinct MV groups could be differentiated: indirect MV (N = 13), abstract MV (N = 11), augmented reality MV (N = 9), avatar MV (N = 5), tracking MV (N = 4), combined MV (N = 1), and no MV (N = 2). In two included articles the visualisation conditions included different MV groups within the same study. Additionally, differences in motor performance could not be analysed because of the differences in the study design. Three studies investigated different visualisations within the same MV group and hence limited information can be extracted from one study.

**Conclusions:**

The review demonstrates that individuals’ movements during VR-based motor training can be displayed in different ways. Future studies are necessary to fundamentally explore the nature of this VR information and its effect on motor outcome.

## Background

Virtual reality (VR) in neurorehabilitation has emerged as a fairly recent approach that shows great promise to enhance the integration of virtual limbs in one`s body scheme [1] and motor learning in general [2]. *Virtual Rehabilitation* is a “group [of] all forms of clinical intervention (physical, occupational, cognitive, or psychological) that are based on, or augmented by, the use of Virtual Reality, augmented reality and computing technology. The term applies equally to interventions done locally, or at a distance (tele-rehabilitation)” [3]. The main objectives of intervention for facilitating motor learning within this definition are to (1) provide repetitive and customized high intensity training, (2) relay back information on patients’ performance via multimodal feedback, and (3) improve motivation [2, 4]. VR therapies or interventions are based on real-time motion tracking and computer graphic technologies displaying the patients’ behaviour during a task in a virtual environment.

The interaction of the user and Virtual environment can be described as a perception and action loop [5]. This motor performance is displayed in the virtual environment and subsequently, the system provides multimodal feedback related to movement execution. Through external (e.g. vision) and internal (proprioception) senses the on-line sensory feedback is integrated into the patient’s mental representation. If necessary, the motor plan is corrected in order to achieve the given goal [5].

A previous Cochrane Review from Laver, George, Thomas, Deutsch, and Crotty [2] on Virtual Reality for stroke rehabilitation showed positive effects of VR intervention for motor rehabilitation in people post-stroke. However, grouped analysis from this review on recommendation for VR intervention provides inconclusive evidence. The author further comments that “[…] virtual reality interventions may vary greatly […], it is unclear what characteristics of the intervention are most important” ([2], p. 14).

Virtual rehabilitation system provides three different types of information to the patient: movement visualisation, performance feedback and context information [6]. During a motor task the patient’s movements are captured and represented in the virtual environment (movement visualisation). According to the task success, information about the accomplished goal or a required movement alteration is transmitted through one or several sensory modalities (performance feedback). Finally, these two VR features are embedded in a virtual world (context information) that can vary from a very realistic to an abstract, unrealistic or reduced, technical environment.

Performance feedback often relies on theories of motor learning and is probably the most studied information type within VR-based motor rehabilitation. Moreover, context information is primarily not designed with a therapeutic purpose. Movement observation, however, plays an important role for central sensory stimulation therapies, such as mirror therapy or mental training. The observation or imagination of body movements facilitates motor recovery [7–9] and provides new possibilities for cortical reorganization and enhancement of functional mobility. Thus, it appears that movement visualisation may also play an important role in motor rehabilitation [10–12], although this aspect is yet to be systematically investigated [13].

The main goal of the present review is to identify various movement visualisation groups in VR-based motor interventions for lower extremities, by means of a systematic literature search. Secondarily, the included studies are further analysed for their effect on motor learning. This will help guide future research in rehabilitation using VR.

An interim analysis of the review published in 2013 showed six MV groups for upper and lower extremity training and additional two MV groups directed only towards lower extremity training. In this paper, we analysed only studies involving lower limb training, leading to a revision and expansion of the previously published MV groups findings [13–15].

## Methods

An electronic search of published literature was performed based on the same keywords and search string as outlined by a previous Cochrane review [2] on virtual reality for stroke rehabilitation. Following databases were searched for relevant studies: MEDLINE (Ovid), AMED, and EMBASE databases were searched from inception to 21st November 2012, and the CINHAL, and PsycInfo databases were searched from inception to 4th December 2012.

Inclusion criteria were as follows: (1) experimental studies on motor rehabilitation for lower extremities, (2) VR visual feedback, (3) healthy participants or any neurological patient population, (4) at least one motor outcome reported. Exclusion criteria were (1) studies with non-virtual environments, and (2) applications focussing to train cognitive functions or psychotherapy. Also publications in languages other than English or German as well as publications or conference abstracts that did not contain sufficient information for the analyses were not included. Since the main goal of this review is to determine and compare various types of movement visualisations there was no exclusion based on the methodological quality of studies.

All search results were exported to bibliographic software (Citavi 4.1, Swiss Academic Software GmbH). One review author (LFS) screened paper titles retrieved from the search in order to exclude obviously irrelevant references. Abstracts or full texts or both of the remaining studies were obtained and used on the inclusion criteria to assess whether they were eligible for inclusion. Disagreements were resolved by discussion between two review authors (LFS and KM). The included articles were first analysed on their use of Movement Visualisation. This was done on the basis of the information provided in the article text and available images. Thereafter, study characteristics were then extracted in a tabular format which included: motor function, sample population, sample size, immersion, and use of robotic device. Immersion was categorized accordingly to Kalawsky [16] into non-immersive (desktop monitor), semi-immersive (large screen monitor or projection systems with more than 60° wide angle display, with or without 3D shutter glasses), and fully immersive (360° wide angle display, e.g. with a Head Mounted Display). Additionally, categories of augmented reality (real world supplemented with virtual information) and commercial gaming system (systems that have not been designed for rehabilitation purposes) were used.

## Results

A total of 4240 articles were identified from the electronic search of which 44 studies were selected based on the inclusion and exclusion criteria. Table 1 gives an overview of all included studies.

**Table 1:** Overview of included studies

Study	Movement Visualisation	Immersion	Robotic device	Motor function	Sample population	Sample size
Aiello [35]	Indirect MV: OF	Non	No	Gait	T: MS C: –	T: 10 C: –
Cikajlo [36]	Indirect MV: OF	Non	No	Balance	T: Stroke C: Stroke	T: 6 C: 22
Fung [37]	Indirect MV: OF	Semi	No	Gait	T: Stroke C: –	T: 2 C: –
Fung [38]	Indirect MV: OF	Semi	No	Gait, Balance	T: Stroke C: healthy	T: 9 C: 9
Kizony [39]	Indirect MV: OF	Semi	No	Gait	T: Stroke C: healthy	T: 12 C: 10
Park [40]	Indirect MV: OF	Semi	No	Gait	T: PD C: –	T: 3 C: –
Yang [41]	Indirect MV: OF	Semi/full	No	Gait	T: Stroke C: Stroke	T: 11 C: 9
Yang [42]	Indirect MV: OF	Non	No	Balance	T: Stroke C: Stroke	T: 7 C: 7
Bergmann [22]	Indirect MV: OF	Non/semi	Yes (Lokomat)	Gait	T: Stroke C: –	T: 1 C: –
Walker [43]	Indirect MV: AOF	Non/semi	No	Gait	T: Stroke C: –	T: 6 C: –
Lamontagne [31]	Indirect MV: AOF	Full	No	Gait	Crossover: (T) Stroke, (C) healthy	Crossover: (T) 12, (C) 12
Lamontagne [44]	Indirect MV: AOF	Full	No	Gait	Crossover: (T) Stroke, (C) healthy	Crossover: (T) 10, (C) 11
Slobounov [24]	Indirect MV: AOF	Semi	No	Balance	T: healthy → TBI (within) C: healthy	T: 10 C: 45
Betker [45]	Abstract MV: 2D	Non	No	Balance	T: Ataxia, Stroke, TBI C: –	T: 3 C: –
Geiger [46]	Abstract MV: 2D	Non	No	Balance	T: Stroke C: Stroke	T: 7 C: 6
Gil-Gomez [47]	Abstract MV: 2D	Non/semi	No	Balance	T: Stroke, TBI, BCN C: Stroke, TBI, BCN	T: 9 C: 8
Jobst [48]	Abstract MV: 2D	Non	No	Balance	T: Ataxia C1: Ataxia C2: healthy	T: 36 C1: 36 C2: 10
Mercier [49]	Abstract MV: 2D	Non	No	Gait	T: Stroke C: –	T: 1 C: –
Forrester [50]	Abstract MV: 2D	Non	Yes (Anklebot)	Ankle	T: Stroke C: –	T: 8 C: –
Cho [17]	Abstract MV: 3D & Avartar MV: Rough figure	Game	No	Balance	T: Stroke C: Stroke	T: 11 C: 11
Deutsch [51]	Abstract MV: 3D	Non	Yes (Rutgers Ankle Rehabilitation System)	Ankle	Exp. 1 & 2: T: Stroke C: –	Exp.1: T: 1, C: – Exp.2: T: 3, C: –
Deutsch [52]	Abstract MV: 3D	Non	Yes (Rutgers Ankle Rehabilitation System)	Ankle	T: Stroke C: –	T: 6 C: –
Mirleman [53, 54]	Abstract MV: 3D	Non	Yes (Rutgers Ankle Rehabilitation System)	Ankle	T: Stroke C: Stroke	T: 9 C: 9
Cattaneo [55]	Tracking MV	Non	No	Balance	T: MS C: –	T: 9 C: –
Deng [56]	Tracking MV	Non	No	Ankle	T: Stroke C: Stroke	T: 8 C: 8
Dunning [57]	Tracking MV	Non	No	Gait, Ankle	T: Stroke C: –	T: 1 C: –
Durfee [58]	Tracking MV	Non	No	Ankle	T: Stroke C: –	T: 20 C: –
McClanachan [59]	Avartar MV: Rough figure	Game	No	Gait, Balance	Crossover: Stroke, TBI	Crossover: 21
Brütsch [18]	Avatar MV: Realistic body	Non/semi	Yes (Lokomat)	Gait	Crossover: (T) BS-CP, TBI, MMC, SLE, (C) healthy	Crossover: (T) 10, (C) 14
Schuler [19]	Avatar MV: Realistic body	Non/semi	Yes (Lokomat)	Gait	Crossover: (T) MS, CP, Hip dysplasia, Cerebral haemorrhage, Encephalopathy, Transverse myelitis, (C) healthy	Crossover: (T) 9, (C) 8
Brütsch [60]	Avatar MV: Realistic body	Non/semi	Yes (Lokomat)	Gait	Crossover: (T) MS, CP, Hip dysplasia, Cerebral haemorrhage, Encephalopathy, symptomatic SCI, (C) healthy	Crossover: (T) 10, (C) 8
Baram [25]	AR MV	AR	No	Gait	T1: MS T2: MS	T1: 10 T2: 11
Baram [61]	AR MV	AR	No	Gait	T: MS C: healthy	T: 16 C: 12
Kim [62]	AR MV	AR	No	Gait, Balance	T: Stroke C: Stroke	T: 12 C: 12
Palma [63]	AR MV	Game	No	Balance	T: TBI C: TBI	T: 7? C: 7?
Sveistrup [64]	AR MV	AR	No	Balance	T1: TBI T2: TBI C: TBI	?
Jaffe [65]	AR MV	AR	No	Gait	T: Stroke C: Stroke	T: 10 C: 10
Thornton [66]	AR MV	AR	No	Balance	T: TBI C: TBI	T: 15 C: 12
Griffin [20]	AR MV	AR	No	Gait	T: PD (within)	T: 26
Ferrarin [21]	AR MV & Combined MV: AR + Abstract (2D)	AR	No	Gait	T: PD (within) C: healthy (within)	T: 3 C: 3
Walker [67]	No MV	Non	No	Balance	T: Stroke C1: Stroke C2: Stroke	T: 16 C1: 16 C2: 14
Banz [68]	No MV	Non	Yes (Lokomat)	Gait	Crossover: iSCI	Crossover: 12

*AR* augmented reality, *OF* optical flow, *AOF* active optical flow, *non* non-immersive, *semi* semi-immersive, *full* full-immersive, *game* commercial game, *Ankle* ankle movement training, *T* treatment group, *C* control group, *MS* multiple sclerosis, *(BS-)CP* bilateral spastic cerebral palsy, *TBI* traumatic brain injury, *BCN* Benign cerebral neoplasm, *(i) SCI* (incomplete) spinal cord injury, *PD* Parkinson’s disease, *MMC* meningomyelocele, *SLE* systemic lupus erythematodes, *healthy* no neurological disorder

### Categorisation of movement visualisation groups

The analyses of the visual information in the 44 included articles led to a differentiation of seven movement visualisation groups: indirect MV, abstract MV, augmented reality (AR) MV, avatar MV, tracking MV, combined MV, and no MV (shown in Table 2).

**Table 2:** Movement Visualisation (MV) Groups

MV Group	N	Description
Indirect MV	13	Body movements are not directly visualized in the virtual environment. Changes in context information represent indirectly the user’s movement
		Subgroups
		Optical flow (N = 11): Motion of patterns or objects create a *naturalistic* illusion of movement in the virtual environment. Goal is usually navigation through a virtual scenario (e.g. supermarket)
		Active optical flow (N = 3): Motion of patterns or objects during movement in the virtual environment is manipulated with the purpose to influence the user’s behavior (e.g. acceleration of optical flow)
Abstract MV	11	The user’s movement is represented in a (non-anthropomorphic) computer graphic. Main goal relies on the correct execution of the task
		Subgroups
		2D Exercise (N = 6): tasks performance in a two dimensional environment
		3D Exercise (N = 5): task performance in a three dimensional environment
Augmented reality (AR) MV	9	Visualisation of the user’s real body supplemented with virtual Performance Feedback and/or virtual Context Information (e.g. Sony Eye Toy, AR-glasses). This also includes Augmented Virtuality
Avatar MV	5	Real movements are represented through a virtual body (or body part)
		Subgroups
		Realistic body (or body part) (N = 3): Representation visually and kinematically close to the real body
		Rough figure (N = 2): Simple body representation with some aspects of real appearance and movements (e.g. Mii avatar in Nintendo Wii)
		Mirrored body (or body part) (N = 0): Realistic body representation with mirrored visual and movement information
Tracking MV	4	Continuous adjustment of body movement with an external visual input (e.g. follow a given trajectory). Explicit goal is the correctness of the movement execution
Combined MV	1	Visualisation consists of more than one MV type (e.g. indirect MV on an augmented reality device)
No MV	2	Body movements are not represented in the virtual environment. Visualisation during rehabilitation training is exclusively based on Performance Feedback

There were several studies that needed careful analysis of the MV to be correctly grouped. The study from Cho et al. [17] was grouped twice, into the MV groups Avatar and Abstract groups, because the authors used several different balance games. They employed the Nintendo Wii balance board with either visualisations of the participants’ body movements through an avatar figure (Mii avatar of the Nintendo Wii) or abstract two- or three-dimensional games without an avatar.

Considering only the information provided in the included paper from Brütsch et al. [18] the two visualisation conditions within the described study seemed to fall within the visualisation groups Avatar MV and Indirect MV. However, the authors of this review were personally informed by the research group that in both conditions the body movements of the participants were represented as an avatars as in a related publication of the same research group [19].

Moreover, the study from Griffin et al. [20] was classified into augmented reality group alone. In this article, 26 participants with Parkinson’s disease induced mobility issues walked through a corridor while receiving information via AR glasses adding visual stimuli to the real environment. The visual conditions included (a) a control condition with VR glasses showing a static image of a rectangle, (b) stripes simulating optical flow (further divided as coherent and reversed visual flow), (c) a rhythmic cueing stimulus transmitting red and black full-screen flashing, and (d) a control condition without VR glasses showing transverse lines on the floor. Neither of the optical flow condition, nor the rhythmic cueing stimulus was synchronised to walking speed or step rhythm. Therefore, the only information on the participants body movements provided was the image of the real body through the colourless lenses.

Also, the study by Ferrarin et al. [21] presented different optical flow stimuli. In this paradigm, the AR glasses drifted forward and backward. Similar to the study from Griffin at el. [20], the rhythm of this visual information was not synchronised to walking speed or step rhythm and hence it could not be classified as an MV. However, there was additional experimental condition in which a visual stimulus was applied on the side of the glasses of the leg that was right before the next step. This attentional signal was activated through data recorded from a foot-switch on the contralateral foot of the signal side, indicating the end of the swing phase of the contralateral leg. This information was classified as an abstract MV and thus the paper was grouped twice into the augmented reality group for the experimental conditions with optical flow stimuli and into combined MV with an augmented reality visualisation of the real body. The latter was presented together with abstract two-dimensional MV information of the movement execution for the foot-switch condition.

In the study by Bergmann and co-workers [22], the classification of the MV was not clear. In this case the study presented a virtual scenario during a robot-assisted walking task to a participant with a hemiparesis following stroke. The visualisation consisted of an active optic flow in form of a moving virtual forest along a red dotted walking path; that moved faster or slower synced to patient’s movement effort within the robotic device. Also a walking dog was presented and the goal of the task was to keep the movement effort as high as necessary for the dot to stay under the dog. In this case the body´s movement was represented both an indirect movement of the virtual scene and an abstract visualisation in form of a dot. In this review, the applied MV was categorised into the Indirect MV group because the visualisation of the dot was mainly applied as an instrument of the performance feedback within the walking task. Nevertheless, it would also be possible to consider the visualisation as combined MV with an indirect and an abstract MV.

One study by Sloubounov et al. [23], which was categorized into indirect MV in previously published findings [14, 15] had to be excluded following detailed consideration. The visualisation applied in this study showed that it was not a MV under consideration. Twelve healthy volunteers performed baseline static standing for 3 blocks of eight conditions on a force platform with an assessment of kinematic through flock of birds motion analysis system and goniometer positioned at right ankle. A black and white striped room was displayed 3D stereo virtual field and synchronized with the motion tracking system. The variation of the conditions consisted of anterior-posterior oscillations of the whole room, the front wall or the side wall at 0.3 or 0.6 Hz. Also, a whole room lateral roll at 0.3 and 0.6 Hz was performed. In a second experiment, the participants were exposed to the same virtual room motion conditions via mirror mounted on a head coil in supine position in an fMRI scanner. Since the participant had the instruction to stand “as steady as possible” ([23], p. 189) and the induced change in kinematics through a simulation of room movement was not linked to a change of visualisation, the visual information in this experiments is better to be classified as a manipulation in Context Information rather than an application of Indirect MV of (active) optical flow. Moreover, no performance feedback was given so that there was no information on body movement given at all.

### Movement visualisation comparison

Most of the included studies did not have their main goal on exploring the effect of MV on motor learning. Instead they addressed to investigate the effect of a VR motor intervention in general. Only a few studies allowed to extract information about the influence of different MV on motor learning, even though it was not the focus of the referring studies. Therefore subsequently we summarise the extracted relevant information. In two studies two MV-groups Avatar and Abstract [17] and augmented reality and combined MV [21] were applied within the same game intervention. Three studies investigate different visualisations within a same MV group [20, 24, 25].

In a second included paper by Sloubonov et al. [24], a comparison of different MVs was performed within one study. In this study, 55 University athletes at risk for traumatic brain injury underwent two experiments with balance force plates and a motion analysis system in front of a 3D wall screen projection. Three follow up testing were conducted with 10 participants which suffered traumatic brain injury (mild concussion) after the baseline recording. The first experiment was comparable with the previous mentioned study by the research group [23]. The participants had to stand as still as possible while applying virtual scenes of moving black and white stripes simulating visual field motion in three different conditions. During the second experiment subjects produced whole body postural movements in forward–backward and lateral directions while viewing visual field randomised across 3 sub-conditions: (1) dark, (2) moving room matched at subject’s body motion at 180° in-phase, and (3) moving room matched at subject’s body motion at 180° out-of-phase. Importantly the variations in active manipulation of the virtual scene (condition 2 and 3) represent a direct comparison of the subgroups “optical flow” (optical flow in accordance to natural movements) and “active optical flow” (manipulated optical flow, in this case conflicting with natural movements) of Indirect MVs within our classification. The analysis of centre of pressure measure and coherence measure based on cross-spectra estimate of the weight shifts showed for the second experiment in all subjects prior to concussion, a preserved balance i.e. coherent oscillatory postural movements that matched virtual room motion, for both sub-condition 2 and 3. Post-injury a decrease in coherence was present at day 10 and day 30. The participants presenting a mild concussion were not able to stay in balance while presenting a conflicting active optical flow. In contrast, subjects post-injury showed a decrease in coherence on day 3 which improved by day 10 and returned to baseline by day 30 during the task with no external manipulation of the virtual scene (experiment 1).

As mentioned previously Ferrarin and colleagues [21] applied augmented reality and combined MV of augmented reality and abstract visualisation in different experimental conditions within the same study. However, a direct comparison of the MV is not possible because the context information varied highly between the conditions. Only during the augmented reality MV an augmented visual field motion was presented whereas in the combined MV task the context information consisted of exclusively the real environment.

Two additional studies [20, 25] compared the applied visual information within the augmented reality group in subjects with Parkinson’s disease and multiple sclerosis. In both studies it was possible for the participants to view their body moving in real-time through the real environment augmented with geometrical patterns. But the visual feedback of the movement per se was not changed. Therefore, the results of these studies could not be considered to provide any guideline for the application of MV, since this information was kept constant across the settings.

## Discussion

In this systematic literature review 43 publications on application of VR-based therapy for lower extremity motor rehabilitation in neurological population, were analysed for their movement visualisation. Seven distinct groups implementing user’s movement into the virtual environment were differentiated as: indirect MV, abstract MV, augmented reality MV, avatar MV, tracking MV, combined MV, and no MV. Unfortunately, no included study directly compared MV groups. Therefore, no conclusion on potential difference on motor learning based on MV groups can be driven.

The visualisation of optical flow or active optical flow (indirect MV) was the most frequently applied and investigated MV for rehabilitation of gait and balance. Only within this MV group diverse forms of visualisations could be analysed and compared.

In addition to visual cues and complex scenes, movement of the body in space continuously generates an optic flow field [26]. The optic signals provide information on direction of locomotion [27] and can modulate speed, in addition to adapting locomotion to the uneven terrain and avoid obstacles [28, 29]. Furthermore, alteration in optic flow speed in a virtual environment is shown to modulate gait speed in healthy subjects [30], with faster optic flow resulting in decrease of walking speed in stroke individuals [31]. Thus, this visual stimulation, via complex scenes or optic flow, can affect locomotion.

In a previously published analysis on VR-based neurological movement rehabilitation for the *upper* extremity, the most applied MV group was the use of an Avatar, in which the person's arm and hand movements were proportionally represented in the virtual environment [13, 14]. This may not be a coincidence. Differences in MV selection between upper and lower extremities applications suggest that further research needs to be conducted on direct visualisation for the upper extremity and indirect visualisation for the lower extremities. Although there is no evidence that direct and indirect display of a user’s movement has an influence on motor outcome it is possible that there is a difference in effectiveness between MV of upper and lower extremities. This could be related to the visual information received in normal motor execution. Upper extremities are used independently for a single or bilateral activity. In contrast, lower limbs are most commonly used for locomotion. Studies have shown that locomotion seems to rely more on “central pattern generators”, integrating rhythmic sensorimotor information with information on body localisation rather than visual information about the position of the lower limbs in space [32]. In contrast, the majority of humans’ tasks with arms and hands require visual information on the body related to the environment, e.g. for grasping an object correctly [33]. Therefore, it may be more effective to train lower extremities with indirect MV showing the movement of the environment according to the displacement of the body during walking without a direct presentation of the limbs. In contrast, motor learning of hand and arm movements may need more information on how exactly the movement of the extremity looks like in relation to the environment.

As mentioned above there is currently insufficient evidence for any concrete conclusion. The tendency, however, gives a reason to expand research on similarities and differences in MV on motor learning of upper and lower extremities. The analysis of included papers on MV comparison showed that only one included paper reported an appropriate study design for this purpose. The study from Sloubonov et al. [24] showed a persistent disturbance in balance for participants with traumatic brain injuries until 30 days after injury only in the active (conflicting) optical flow condition and not for the natural optical flow condition. Moreover, these findings were not observed for the healthy individuals. Based on this, the authors concluded that VR applications can be used to examine the effect on balance and “potentially be considered within the scope of existing grading scales of concussion” ([24], p. 191). Nevertheless, it has to be summarised that there is insufficient evidence to conclude about different effects of the several MV groups on walking or balance.

The current systematic review is limited in some aspects. First, only VR applications aimed at rehabilitation of neurologically impaired population were included. It is possible, however, that VR-based systems promote motor learning in healthy individuals or people with orthopaedic restrictions, requiring other MVs. To our understanding and knowledge, it is currently unclear if there is a difference of MV on motor learning among these populations. Another limitation is that rehabilitation of neuro-cognitive dysfunctions may have importance for an optimal MV design. Moreover, there may be an interaction of movement visualisation with performance feedback and context information on motor learning. For example, the same movement visualisation probably has another effect when combined with knowledge of results (KR) than with knowledge of performance (KP), both performance feedback components, and vice versa. This study does also not conclude on possible effects or interactions of information from other modalities like sound or haptic feedback, which was provided in the included studies. Future studies should explore the relationship of VR information components in several modalities on human behaviour and motor outcome. Finally, there were several included studies that applied robotic technologies. A previous study compared the effects of VR application alone in contrast to a passive exoskeleton and an end effector haptic device on upper extremity training in post-stroke participants [34]. All conditions showed increased clinical outcome measurements after the trial with no significant difference between different technical applications. However, only in the haptic feedback condition the achieved gains in ability were retained to the follow up assessment 12 weeks post-intervention [34]. In this study the virtual environment was kept constant for all three applications. The results suggest that there may be different motor learning strategies involved when a patient is assisted in movement execution. Therefore, potential interaction of MV with robotic devices should be considered in future research.

Today a vast number of VR-based rehabilitation applications are available. Most of them are effective, efficient and provide an additional tool to conventional therapy [2]. However, there is lack of knowledge on the mechanism of effect with use of these interventions. This makes the creation of new interventions possibly inefficient with increase in cost for the evaluation. Information on how virtual rehabilitation features influence motor learning is necessary and should provide important insight in how these features need to be modified according to the therapeutic goal.

The results of this review are a first step on systemizing VR-based motor rehabilitation applications. The MV groups showed how diverse VR training can be implemented and the limited knowledge about the effect of these distinct visualisations. Based on the presented MV groups in this review, future research could be carried out focusing on and investigating the effect of diverse visualisation on motor outcome and other rehabilitation related aspects such as motivation and presence [6].

## Conclusions

This study was performed in order to categorize and analyse movement visualisation of current available literature on lower extremity VR-based motor neuro-rehabilitation. In the systematic literature search it was possible to describe seven distinct groups of MV indicating that movements are currently displayed very differently in virtual rehabilitation environments. Unfortunately, little is known about the effect of different MVs on motor outcome. There is not enough information on how different MV influences motor learning within the included studies so that no recommendation on MV for specific purpose of interventions can be deduced at this stage. Additional research in is necessary to optimize future interventions.

## Abbreviations

VR: virtual reality
MV: movement visualisation
AR: augmented reality
